# Sex modifies association between dietary sodium intake and cardiovascular disease mortality among US adult with hypertension: a national population-based cohort

**DOI:** 10.3389/fcvm.2024.1471647

**Published:** 2024-11-28

**Authors:** Zhiqiang Chen, Jingan Rao, Weiguo Fan, Zuxiang Wu, Yumeng Shi, Yingxing Wu, Huan Hu, Xiaoshu Cheng, Ping Li

**Affiliations:** ^1^ Department of Cardiovascular Medicine, The Second Affiliated Hospital of Nanchang University, Nanchang, Jiangxi, China; ^2^ Department of Cardiovascular Medicine, The First Affiliated Hospital of Gannan Medical University, Ganzhou, Jiangxi, China

**Keywords:** CVD, mortality, hypertension, dietary sodium intake, female, National Health and Nutrition Examination Surveys

## Abstract

**Objectives:**

The objective of this study was to examine the relationship between dietary sodium intake and cardiovascular disease (CVD) mortality in hypertensive American adults.

**Methods:**

A prospective cohort study was conducted to examine the association between dietary sodium intake, as estimated by a single 24-h dietary recall from the National Health and Nutrition Examination Survey (2003–2012), and mortality data obtained from the National Death Index.

**Results:**

This study included 12,236 adults with hypertension, with 837 CVD-related deaths identified over a median follow-up period of 10.3 years. A nonlinear association between dietary sodium intake and CVD mortality was observed. The inflection point of the curve occurred at a sodium intake level of 2.07 g/day. Below this threshold, higher sodium intake was associated with a reduced risk of CVD mortality, though the association was not statistically significant after full adjustment (aHR: 0.78, 95% CI: 0.58–1.05). In contrast, sodium intake above 2.07 g/day was significantly associated with an increased risk of CVD mortality (aHR: 1.12, 95% CI: 1.02–1.23). The log-likelihood ratio test yielded a *P*-value of 0.04. This J-shaped association was observed exclusively in females, not males. Among females, the adjusted hazard ratios (95% CI) were 0.65 (0.42, 0.99) below and 1.29 (1.11, 1.53) above the inflection point (*P* for log-likelihood ratio test = 0.009).

**Conclusions:**

In American adults with hypertension, dietary sodium intake exceeding 2.07 g/day was significantly associated with an increased risk of CVD mortality, while intake below this threshold was not significantly linked to mortality risk. Additionally, a sex-specific effect of dietary sodium intake on CVD mortality was observed, showing a J-shaped relationship exclusively in females, with no such association found in males.

## Introduction

Hypertension is a significant risk factor for cardiovascular disease (CVD) which continues to be the leading cause of death globally (1). Clinical guidelines from respected organizations such as World Health Organization (WHO) (2), the American Heart Association (3) and the European Society of Cardiology (4) stress the importance of reducing sodium intake to below 2 grams per day (g/day) to lower blood pressure and decrease the risk of CVD.

However, previous prospective studies have reported conflicting findings regarding the relationship between sodium consumption and the risk of CVD events (5). Some studies indicated a positive correlation between sodium intake and CVD mortality, suggesting that higher sodium intake may elevate the risk of such events. Conversely, other research has found no significant association between sodium intake and CVD mortality (6, 7), and some have even reported an inverse relationship (8–11), where higher sodium intake appears to be linked to lower mortality rates. Additionally, there is a notable gap in research examining the impact of sodium intake on CVD mortality specifically among adults diagnosed with hypertension.

To address these research gaps, we conducted a prospective study to examine the relationship between dietary sodium intake and the risk of CVD mortality in a sample of US adults with hypertension that is representative of the entire population.

## Methods

### Participants

The National Health and Nutrition Examination Survey (NHANES) is an ongoing survey conducted by the Centers for Disease Control and Prevention (CDC). Employing a complex sampling methodology, NHANES selects participants from diverse age groups, ethnicities, and socioeconomic backgrounds to gather comprehensive data on various health and nutrition factors through interviews, physical examinations, and laboratory tests. This survey plays a critical role in shaping public health policy, monitoring health trends, and advancing scientific research. Additional details about NHANES can be found on its official website.

**Figure 1:**
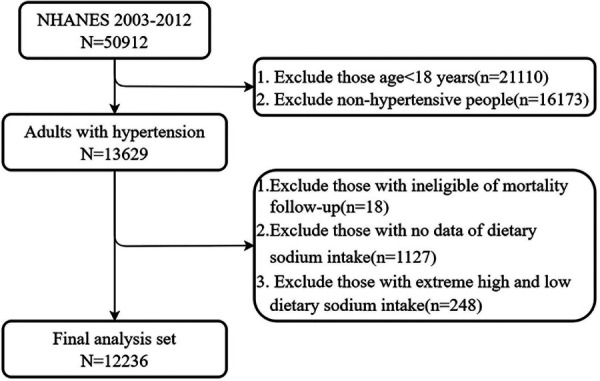
Flow chat of participant selection.

As shown in Figure 1, adults aged 18 years and older with hypertension from five consecutive NHANES cycles (NHANES 2003–2012) were included in the study. A total of 13,629 participants were diagnosed with hypertension, defined as having a systolic blood pressure (SBP) of ≥130 mmHg, a diastolic blood pressure (DBP) of ≥80 mmHg, or self-reported hypertension. After excluding participants lost to mortality follow-up and those with missing sodium intake values, 12,484 participants with hypertension remained. Data for sodium intake above the 99th percentile and below the 1st percentile were excluded to eliminate extremely high and low values. Ultimately, 12,236 participants were included in the analysis.

### Dietary sodium intake

Dietary sodium intake was derived from the NHANES dietary interview. This component of NHANES gathers comprehensive information on dietary consumption and estimates energy, nutrients, and other food components from the foods and beverages consumed in the 24 h prior to the interview. This information is collected through structured in-person dietary interviews conducted at the mobile examination center, utilizing the Automated Multiple Pass Method—a validated approach for accurate dietary recall (12).

### CVD mortality

The mortality status of the follow-up population was obtained from the NHANES public-use linked mortality file, as of December 31, 2019, which is connected to the National Death Index (NDI). To identify deaths related to cardiovascular disease (CVD), we utilized the International Statistical Classification of Diseases, 10th Revision (ICD-10). This classification system provides standardized coding and classification for diseases, including CVD, with specific codes ranging from I00 to I09, I11, I13, and I20 to I51.

### Co-variables

Demographic information such as age, sex, race/ethnicity, educational level, and medical history, including medication use, was collected through interviews. Educational attainment was categorized into three groups: college or higher, high school, and less than high school. Smoking status was classified as current smoker, former smoker, and never smoker. Alcohol consumption was divided into three categories: none (0 g/day), moderate (0.1–27.9 g/day for men and 0.1–13.9 g/day for women), and heavy (≥28 g/day for men and ≥14 g/day for women). Body mass index (BMI) was calculated by dividing weight (kg) by height squared (m^2^), with obesity defined as BMI ≥30 kg/m^2^. Blood pressure (BP) was measured three consecutive times, and the mean value of these measurements was used in the analysis. Diabetes was diagnosed based on a glycosylated hemoglobin (HbA1c) level of ≥6.5%, fasting plasma glucose ≥126 mg/dL, or self-reported diabetes diagnosis. Clinical parameters measured in the NHANES laboratory included serum sodium, serum potassium, aspartate aminotransferase (AST), alanine aminotransferase (ALT), uric acid, serum creatinine, triglycerides, total cholesterol (TC), high-density lipoprotein cholesterol (HDL-C), and HbA1c. The estimated glomerular filtration rate (eGFR) was calculated using the CKD-EPI equation (13).

### Statistical analysis

Given the complex sampling design of the NHANES database, we incorporated sample weights, clustering, and stratification into our data analyses in accordance with NHANES analytic guidelines. Continuous variables were expressed as means with standard deviations (SD), while categorical variables were presented as counts (percentages). Baseline characteristics were compared using one-way analysis of variance (ANOVA) for continuous variables and chi-square tests for categorical variables.

The relationship between sodium intake and cardiovascular mortality was evaluated using a Cox proportional hazards model for the overall population as well as separately for males and females. Model 1 was adjusted for age. In Model 2, adjustments were made for age, sex (for the overall population), race/ethnicity, education level, smoking status, alcohol consumption, diabetes, body mass index (BMI), and potassium intake. Model 3 included adjustments for the following parameters: age, sex (for the overall population), race/ethnicity, education level, smoking status, alcohol consumption, diabetes, BMI, potassium intake, energy intake, systolic blood pressure (SBP), diastolic blood pressure (DBP), cholesterol-lowering medications, glucose-lowering medications, antihypertensive medications, estimated glomerular filtration rate (eGFR), total cholesterol (TC), high-density lipoprotein cholesterol (HDL-C), triglycerides, alanine aminotransferase (ALT), aspartate aminotransferase (AST), and uric acid.

To identify nonlinear relationships between sodium intake and CVD mortality, we employed a Cox proportional hazards regression model with a generalized additive model (GAM) and smooth curve fitting using the penalized spline method. If a nonlinear relationship was detected, a recursive algorithm was applied to calculate the inflection point and select the value with the highest likelihood. Subsequently, a two-piecewise Cox proportional hazards model was constructed on either side of the inflection point (14). To mitigate the possibility of reverse causation, we excluded individuals who reported a history of heart attack, heart failure, or stroke at baseline, and repeated the Cox proportional hazards regression model on this restricted study sample.

All analyses were performed using EmpowerStates (www.empowerstats.com) and R version 4.0.3 (www.R-project.org). A two-tailed *P*-value of <0.05 was considered statistically significant.

## Results

### Characteristics of participants

A total of 12,236 adults with hypertension participated in this study, including 5,898 females and 6,338 males. Among the entire cohort, 5.81% identified as Mexican Americans, 3.59% as other Hispanics, 72.3% as non-Hispanic whites, 13.13% as non-Hispanic blacks, and 5.17% as individuals of other races. Additionally, 16.75% of participants had diabetes, and 44.64% were classified as obese. The average age of the participants was 54.52 years, and their average daily sodium intake was 3.41 grams. The baseline characteristics of the study participants, categorized by sodium intake tertiles, are presented in Table 1. Compared to participants in the lowest tertile of sodium intake, those in the highest tertile were more likely to be male, non-Hispanic white, obese, and either former or current smokers. They also had a higher prevalence of moderate to heavy alcohol use, achieved higher education levels, and exhibited greater values for DBP, BMI, potassium intake, energy intake, triglycerides, eGFR, ALT, AST, and uric acid. Conversely, participants in the highest tertile had a lower prevalence of diabetes, a reduced rate of antihypertensive drug use, and lower rates of glucose-lowering and cholesterol-lowering drug use, as well as lower SBP, TC, HDL-C, and HbA1c. There were no significant differences in serum creatinine, serum sodium, or serum potassium levels across the sodium intake tertiles.

**Table 1:** Baseline characteristic of individuals with hypertension by tertiles of sodium intake in NHANES 2003–2012.

Variables	Whole cohort *N* = 12,236	Tertiles of sodium (g)			*P*
		T1 (<2.37 g) *N* = 4,125	T2 (2.37–3.63 g) *N* = 4,074	T3 (>3.63 g) *N* = 4,037	
Demographic variables					
Age, year	54.52 (0.33)	58.82 (0.39)	55.62 (0.42)	50.24 (0.40)	<0.001
Male sex (%)	6,338 (51.27)	1,519 (33.14)	2,037 (46.37)	2,782 (69.52)	<0.001
Race/Ethnicity (%)					<0.001
Mexican American	1,714 (5.81)	672 (6.60)	524 (5.15)	518 (5.79)	
Other Hispanic	828 (3.59)	315 (4.03)	261 (3.24)	252 (3.56)	
Non-Hispanic White	5,953 (72.30)	1,878 (69.76)	2,051 (72.94)	2,024 (73.70)	
Non-Hispanic Black	3,083 (13.13)	1,100 (15.74)	1,005 (12.78)	978 (11.44)	
Other race	658 (5.17)	160 (3.88)	233 (5.90)	265 (5.51)	
Education level(%)					<0.001
Less than high school	3,646 (19.81)	1,530 (25.18)	1,146 (18.76)	970 (16.62)	
High school	3,023 (26.15)	1,016 (27.21)	1,007 (25.88)	1,000 (25.59)	
College or higher	5,356 (54.03)	1,524 (47.61)	1,860 (55.36)	1,972 (57.79)	
Comorbidities					
Smoking status (%)					0.004
Never	5,990 (49.77)	2,172 (51.82)	2,004 (50.36)	1,814 (47.68)	
Former	3,727 (30.68)	1,212 (30.16)	1,275 (31.29)	1,240 (30.55)	
Current	2,316 (19.55)	692 (18.02)	735 (18.35)	889 (21.78)	
Alcohol intake (%)					<0.001
None	9,408 (72.42)	3,400 (77.86)	3,139 (73.04)	2,869 (67.68)	
Moderate	1,038 (9.24)	268 (6.97)	357 (9.13)	413 (11.07)	
Heavy	1,790 (18.34)	457 (15.17)	578 (17.83)	755 (21.24)	
BMI, kg/m^2^	30.30 (0.10)	29.61 (0.14)	30.15 (0.13)	30.97 (0.18)	<0.001
SBP, mmHg	131.81 (0.29)	133.44 (0.48)	132.66 (0.35)	129.86 (0.37)	<0.001
DBP, mmHg	74.74 (0.31)	72.38 (0.45)	74.72 (0.38)	76.49 (0.36)	<0.001
Diabetes (%)	2,634 (16.75)	992 (18.73)	836 (16.06)	806 (15.85)	0.003
Obesity (%)	5,380 (44.64)	1,726 (40.88)	1,759 (43.82)	1,895 (48.21)	<0.001
Medication use					
Antihypertensive drugs (%)	6,516 (49.13)	2,456 (55.98)	2,172 (49.10)	1,888 (43.91)	<0.001
Glucose-lowering drugs (%)	2,085 (12.08)	806 (14.73)	665 (12.21)	614 (11.85)	<0.001
Cholesterol-lowering drugs (%)	3,379 (29.93)	1,216 (32.52)	1,135 (29.89)	1,028 (27.91)	0.002
Laboratory variables					
Sodium intake, g	3.41 (0.02)	1.75 (0.01)	2.98 (0.01)	5.05 (0.02)	<0.001
Potassium intake, g	2.68 (0.02)	1.93 (0.02)	2.52 (0.02)	3.41 (0.03)	<0.001
Energy intake, kcal/day)	2,103.66 (14.37)	1,362.36 (10.41)	1,941.86 (12.40)	2,815.85 (20.22)	<0.001
Serum sodium, mmol/L	139.11 (0.06)	139.13 (0.07)	139.05 (0.07)	139.15 (0.08)	0.653
Serum potassium, mmol/L	3.99 (0.01)	3.98 (0.01)	3.99 (0.01)	4.00 (0.01)	0.183
TC, mg/dL	201.21 (0.58)	202.55 (0.87)	202.49 (1.03)	199.08 (0.90)	0.004
HDL-C, mg/dL	52.15 (0.24)	54.60 (0.39)	52.99 (0.39)	49.57 (0.32)	<0.001
Triglyceride, mg/dL	174.16 (2.08)	163.53 (3.46)	170.46 (2.62)	185.38 (3.63)	<0.001
HbA1c (%)	5.77 (0.01)	5.81 (0.02)	5.79 (0.02)	5.73 (0.02)	0.001
eGFR, ml·min-1·1.73m-2	85.97 (0.45)	80.85 (0.55)	85.18 (0.60)	90.50 (0.48)	<0.001
Serum creatinine, mg/dL	0.95 (0.01)	0.97 (0.01)	0.93 (0.01)	0.95 (0.01)	0.099
AST, IU/L	26.79 (0.18)	26.48 (0.24)	26.51 (0.40)	27.27 (0.26)	0.022
ALT, IU/L	27.01 (0.23)	24.87 (0.34)	25.97 (0.36)	29.53 (0.48)	<0.001
Uric acid, mg/dL	5.78 (0.02)	5.67 (0.04)	5.70 (0.03)	5.92 (0.03)	<0.001

Data were expressed as means (SD) for continuous variables, and counts (percentage) for categorical variables.

BMI, body mass index; SBP, systolic blood pressure; DBP, diastolic blood pressure; TC, total cholesterol; HDL-C, high density lipoprotein cholesterol; HbA1c, glycosylated hemoglobin; eGFR, estimated glomerular filtration rate; AST, aspartate aminotransferase; ALT, alanine aminotransferase; BUN, blood urea nitrogen.

### Associations of sodium intake with CVD mortality

In this extended mortality linkage study, the median follow-up duration was 10.3 years (IQR: 8.0–12.8 years). During 125,373.5 person-years of follow-up, we identified 837 deaths due to CVD. The independent role of sodium intake in CVD mortality was evaluated using three Cox proportional hazards regression models. As shown in Table 2, when sodium intake was analyzed as a continuous variable using one-piecewise Cox proportional hazards regression, no significant association was found between sodium intake and CVD mortality after adjusting for age [adjusted hazard ratio (aHR): 1.03; 95% confidence interval (CI): 0.98–1.09]. This lack of statistical significance was consistent across all adjusted models: Model 2 (aHR: 1.03, 95% CI: 0.97–1.10) and Model 3 (aHR: 1.06, 95% CI: 0.98–1.15).

**Table 2:** Hazard ratios of CVD mortality by sodium intake among adults with hypertension in NHANES 2003–2012.

Sodium intake	Person-years	Events	CVD mortality rate	HR (95% CI), *P*-value		
			(per year 1000 people)	Model 1	Model 2	Model 3
Per 1 g increase	125,372.50	837	6.68	1.03 (0.98, 1.09), 0.224	1.03 (0.97, 1.10), 0.336	1.06 (0.98, 1.15) 0.147
Inflection point				2.07 g/day	2.07 g/day	2.07 g/day
Sodium intake <2.07 g/day	29,919.75	268	8.96	0.79 (0.63, 0.99), 0.037	0.78 (0.62, 0.99), 0.041	0.78 (0.58, 1.05), 0.104
Sodium intake >2.07 g/day	95,452.75	569	5.96	1.08 (1.02, 1.15), 0.014	1.08 (1.00, 1.16), 0.039	1.12 (1.02, 1.23), 0.020
*P* for log likelihood ratio test				0.018	0.016	0.040

Model 1 was adjusted age, Model 2 adjusted age, sex, race/ethnicity, education level, smoking status, alcohol using, diabetes, BMI, potassium intake; Model 3 adjusted age, sex, race/ethnicity, education level, smoking status, alcohol using, diabetes, BMI, potassium intake, energy intake antihypertensive drugs, glucose-lowering drugs, gholesterol-lowering drugs, SBP, DBP, eGFR, TC, HDL-C, triglyceride, ALT, AST, uric acid.

BMI, body mass index; SBP, systolic blood pressure; DBP, diastolic blood pressure; eGFR, estimated glomerular filtration rate; TC, total cholesterol, HDL-C, high density lipoprotein cholesterol; AST, aspartate aminotransferase; ALT, alanine aminotransferase.

**Figure 2:**
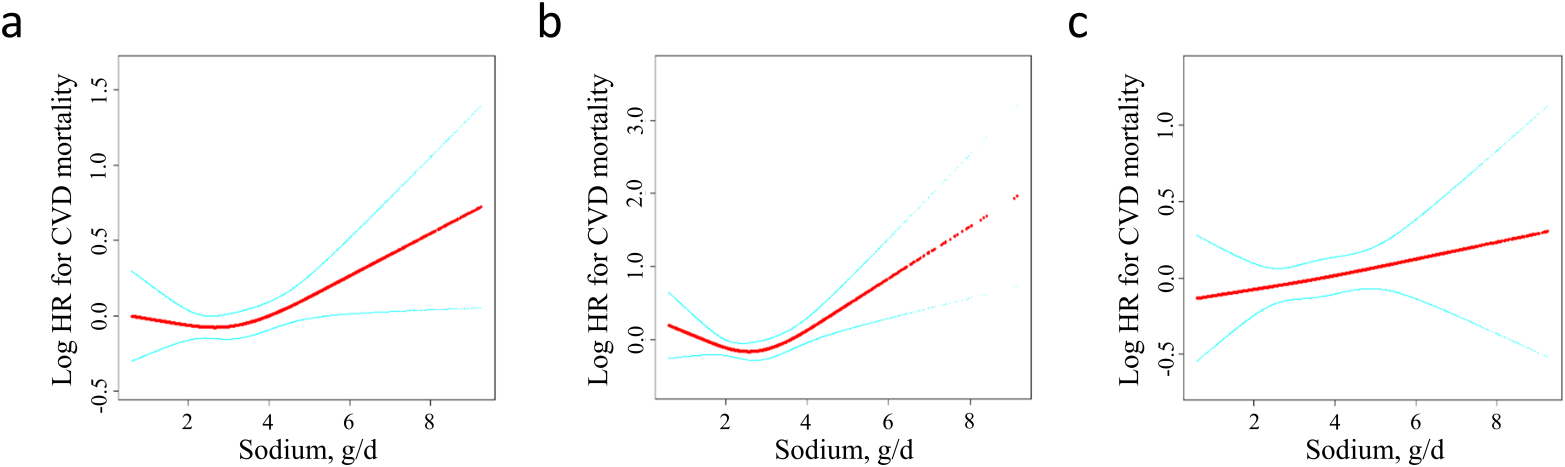
Dose-response analysis between dietary sodium intake and the risk of CVD mortality among **(a)** adults with hypertension **(b)** females with hypertension **(c)** males with hypertension. Adjustment factors included age, sex (only for the overall population), race/ethnicity, education level, smoking status, alcohol using, diabetes, BMI, potassium intake, energy intake, antihypertensive drugs, glucose-lowering drugs, cholesterol-lowering drugs, SBP, DBP, eGFR, TC, HDL-C, triglyceride, ALT, AST, uric acid. BMI, body mass index; SBP, systolic blood pressure; DBP, diastolic blood pressure; eGFR, estimated glomerular filtration rate; TC, total cholesterol; HDL-C, high density lipoprotein cholesterol; AST, aspartate aminotransferase; ALT, alanine aminotransferase.

To analyze the nonlinear association between sodium intake and CVD mortality, we employed a Cox proportional hazards regression model with a generalized additive model (GAM) and smooth curve fitting. As depicted by the smooth curve (adjusted Model 3), sodium intake levels demonstrated a nonlinear relationship with CVD mortality. A Cox proportional hazards regression model with GAM and smooth curve fitting was used to analyze the nonlinear association between sodium intake and CVD mortality. As shown by the smooth curve (adjusted model 3), sodium intake levels are nonlinearly associated with CVD mortality (Figure 2a).

Further analyses were conducted, including a threshold effect analysis and two-piecewise Cox proportional hazards model of sodium on CVD mortality. We determined that the inflection point of the sodium intake was 2.07 g/day by using recursive algorithm. When sodium was <2.07 g/day, increased sodium was associated with reduced risk of CVD mortality (model 1, aHR: 0.79, 95% CI: 0.63–0.99; model 2, aHR: 0.78, 95% CI: 0.62–0.99), but this association was not significant after adjusting for model 3 (model 3, aHR: 0.78, 95% CI: 0.58–1.05). Inversely, When sodium was >2.07 g/day, increased sodium was associated with increased risk of CVD mortality (model 1, aHR: 1.08, 95%CI: 1.02–1.15; model 2, aHR: 1.08, 95% CI: 1.00–1.16; model 3, aHR: 1.12, 95% CI: 1.02–1.23). The log-likelihood ratio test yielded a *P*-value of 0.04, indicating that the two-piecewise Cox proportional hazards model was the more appropriate fit for the association between sodium intake and mortality.

### Associations of sodium intake with CVD mortality stratified by sex

The association between sodium intake and CVD mortality was further examined with a stratification by sex. As showed in Figure 2b, a J-shaped relationship was observed between sodium intake and CVD mortality among females. However, no statistically significant results were found in the one-line Cox proportional hazards model for the female population across all adjusted models: Model 1 [adjusted hazard ratio (aHR): 0.97; 95% confidence interval (CI): 0.88–1.06], Model 2 (aHR: 1.04; 95% CI: 0.93–1.17), and Model 3 (aHR: 1.12; 95% CI: 0.96–1.30). In the analysis using a two-piecewise Cox proportional hazards model, the adjusted hazard ratios in the fully adjusted Model 3 were 0.65 (95% CI: 0.42–0.99) on the left side of the inflection point and 1.29 (95% CI: 1.08–1.53) on the right side. The *P*-value for the log-likelihood ratio test was 0.009 (Table 3).

**Table 3:** Hazard ratios of CVD mortality by sodium intake among female with hypertension in NHANES 2003–2012.

Sodium intake	Person-years	Events	CVD mortality rate	HR (95% CI), *P*-value		
			(per year 1,000 people)	Model 1	Model 2	Model 3
Per 1 g increase	61,359.92	370	6.03	0.97 (0.88, 1.06), 0.514	1.04 (0.93, 1.17), 0.477	1.12 (0.96, 1.30), 0.141
Inflection point				2.07	2.07	2.07
Sodium intake <2.07 g/day	19,732.92	168	8.51	0.67 (0.49, 0.90), 0.008	0.70 (0.51, 0.96), 0.029	0.65 (0.42, 0.99), 0.043
Sodium intake >2.07 g/day	41,627.00	202	4.85	1.08 (0.96, 1.21), 0.221	1.16 (1.01, 1.32), 0.031	1.29 (1.08, 1.53), 0.004
*P* for log likelihood ratio test				0.013	0.011	0.009

Model 1 was adjusted age, Model 2 adjusted age, race/ethnicity, education level, smoking status, alcohol using, diabetes, BMI, potassium intake; Model 3 adjusted age, race/ethnicity, education level, smoking status, alcohol using, diabetes, BMI, potassium intake, energy intake, antihypertensive drugs, glucose-lowering drugs, gholesterol -lowering drugs, SBP, DBP, eGFR, TC, HDL-C, triglyceride, ALT, AST, uric acid.

BM, body mass index; SBP, systolic blood pressure; DBP, diastolic blood pressure; eGFR, estimated glomerular filtration rate; TC, total cholesterol, HDL-C, high density lipoprotein cholesterol; AST, aspartate aminotransferase; ALT, alanine aminotransferase.

In males, no association was found between sodium intake and CVD mortality (Figure 2c). The results from the one-line Cox proportional hazards model indicated that sodium intake does not correlate with CVD mortality: Model 1 [adjusted hazard ratio (aHR): 1.00; 95% confidence interval (CI): 0.94–1.07], Model 2 (aHR: 1.03; 95% CI: 0.95–1.12), and Model 3 (aHR: 1.05; 95% CI: 0.95–1.16). In the analysis using a two-piecewise Cox proportional hazards model, the aHR (95% CI) was 0.99 on the left side of the inflection point and 1.06 (95% CI: 0.95–1.18) on the right side in the fully adjusted Model 3. The *P*-value for the log-likelihood ratio test was 0.789 (Table 4).

**Table 4:** Hazard ratios of CVD mortality by sodium intake among male with hypertension in NHANES 2003–2012.

Sodium intake	Person-years	Events	CVD mortality rate	HR (95% CI), *P*-value		
			(per year 1,000 people)	Model 1	Model 2	Model 3
Per 1 g increase	64,012.58	467	7.29	1.00 (0.94, 1.07), 0.880	1.03 (0.95, 1.12), 0.437	1.05 (0.95, 1.16), 0.335
Inflection point				2.07	2.07	2.07
Sodium intake <2.07 g/day	10,186.83	100	9.82	0.89 (0.62, 1.26), 0.502	0.95 (0.66, 1.36), 0.776	0.99 (0.63, 1.55), 0.961
Sodium intake >2.07 g/day	53,825.75	367	6.82	1.02 (0.94, 1.10), 0.605	1.04 (0.95, 1.14), 0.365	1.06 (0.95, 1.18), 0.326
*P* for log likelihood ratio test				0.482	0.644	0.789

Model 1 was adjusted age, Model 2 adjusted age, race/ethnicity, education level, smoking status, alcohol using, diabetes, BMI, potassium intake; Model 3 adjusted age, race/ethnicity, education level, smoking status, alcohol using, diabetes, BMI, potassium intake, energy intake, antihypertensive drugs, glucose-lowering drugs, gholesterol-lowering drugs, SBP, DBP, eGFR, TC, HDL-C, triglyceride, ALT, AST, uric acid.

BMI, body mass index; SBP, systolic blood pressure; DBP, diastolic blood pressure; eGFR, estimated glomerular filtration rate; TC, total cholesterol, HDL-C, high density lipoprotein cholesterol; AST, aspartate aminotransferase; ALT, alanine aminotransferase.

### Sensitivity analysis

When the analyses were repeated for participants without a reported history of pre-existing cardiovascular disease (CVD), the results remained consistent. Among hypertensive adults without such a history, a nonlinear association was observed between dietary sodium intake and CVD mortality (Supplementary Figure S1A). The one-line Cox proportional hazards model indicated that no statistically significant results were found across all adjusted models [Model 3: hazard ratio (HR): 1.10; 95% confidence interval (CI): 0.99–1.22]. In the two-piecewise Cox proportional hazards model, the adjusted hazard ratio (aHR; 95% CI) was 0.74 on the left side of the inflection point and 1.17 on the right side in the fully adjusted Model 3. The *P*-value for the log-likelihood ratio test was 0.046 (Supplementary Table S1).

Among hypertensive females without a history of pre-existin CVD, a J-shaped association was observed between sodium intake and CVD mortality (Supplementary Figure S1B). Specifically, when sodium intake was less than 2.07 g/day, higher sodium intake was associated with a reduced risk of CVD mortality [Model 3: adjusted hazard ratio (aHR): 0.53; 95% confidence interval (CI): 0.32–0.88]. Conversely, when sodium intake exceeded 2.07 g/day, increased sodium intake correlated with an elevated risk of CVD mortality (Model 3: aHR: 1.39; 95% CI: 1.14–1.70). The *P*-value for the log-likelihood ratio test was 0.046 (Supplementary Table S2).

In contrast, among hypertensive males without a history of CVD, no association was found between sodium intake and CVD mortality (Supplementary Figure S1, Supplementary Table S3).

## Discussion

Using NHANES data from 2003 to 2012, we conducted a large-scale, prospective study that identified a significant nonlinear association between dietary sodium intake and CVD mortality in US adults with hypertension. Our findings revealed that both low and high levels of dietary sodium consumption were linked to an increased risk of CVD mortality in females, whereas no such association was observed in males. The threshold effect analysis indicated that a dietary sodium intake of 2.07 g/day may be considered safe concerning CVD mortality for women. This study is the first to explore the sex differences in the association between dietary sodium intake and CVD mortality among hypertensive individuals.

Several studies have investigated the relationship between sodium intake and CVD mortality in hypertensive patients, yielding mixed results. According to a prospective cohort study involving 3,126 individuals with prehypertension from the Trials of Hypertension Prevention found that higher 24-h urinary sodium excretion—a proxy for sodium intake—was associated with an increased risk of CVD mortality (15). Similarly, another prospective cohort study of 7,543 subjects from the Prevention of Renal and Vascular End-stage Disease study reported a positive association between 24-h urinary sodium excretion and CVD mortality among hypertensive participants (16). Conversely, a prospective cohort study involving 3,505 hypertensive participants found no significant association between 24-h urinary sodium excretion and CVD mortality (17). Additionally, an inverse association between dietary sodium intake and CVD mortality was observed in a prospective study of 8,542 hypertensive individuals, although this finding did not reach statistical significance (7). These contradictory results may be attributable to variations in the characteristics of the cohorts studied, sample sizes, adjustments for confounding variables, and methods used to assess sodium intake. It is also noteworthy that some studies have focused solely on the linear relationship between sodium intake and CVD mortality without considering potential nonlinear associations (7, 16, 17).

The results of this study suggest that both low and high dietary sodium intakes in hypertensive individuals are associated with increased CVD mortality, although some results did not meet statistical significance. Several prospective cohort studies have consistently identified a U-shaped relationship between sodium intake and cardiovascular disease (CVD) or mortality risk, where both high and low sodium intake are linked to adverse outcomes (18–22). This association has been observed across different populations, including individuals with diabetes (18, 19), vascular disease (22), and in the general population (20–22), and has been reported in studies employing various methods for estimating sodium intake (i.e., single 24-h urine collection (18, 19, 22), morning fasting urine samples (20, 21). An analysis of four international prospective studies involving a total of 133,118 individuals (63,559 with hypertension and 69,559 without hypertension) demonstrated a U-shaped association between 24-h urinary sodium excretion—calculated based on fasting morning urine—and CVD mortality among those with hypertension (23). However, for individuals with hypertension who are treated with diuretics to manage blood pressure and exhibit a high prevalence of renal dysfunction, the use of a single overnight urine sample to estimate 24-h urinary sodium excretion as a proxy for daily sodium intake presents a notable limitation (24). Also, the country-specific validated 24-h dietary recall measures and dietary records employed in our study have been shown to underestimate actual sodium intake due to memory bias and day-to-day variability (5). Multiple 24-h urinary sodium excretion measurements are considered the gold standard for estimating daily dietary sodium intake, as they can overcome the accuracy limitations of other methods, such as spot urine samples or dietary recall.

Further analysis of the relationship between dietary sodium intake and CVD mortality by sex revealed a J-shaped association among females, whereas no such association was observed in males. One accepted hypothesis is that the higher risk of CVD mortality among women reach menopause around the age of 50 years old due to hormonal changes in menopause, that is, estrogen consumption (25, 26). Additionally, estrogen deficiency can impact the renin-angiotensin-aldosterone system, which controls blood pressure, increasing the risk of CVD in postmenopausal women (27). Another possible cause is that high salt sensitivity is more common among females (28). A comprehensive and rigorously conducted feeding study revealed that women aged 45 years or older exhibited more pronounced responses in blood pressure (BP), a significant risk factor for CVD mortality, in response to dietary sodium intake (29).

In addition, we identified an inflection point for dietary sodium intake at 2.07 g/day, which closely aligns with existing guidelines for sodium restriction in hypertensive individuals, which generally recommend a daily intake of less than 2 g/day (2–4). However, our findings provide additional nuance by revealing a sex-specific nonlinear association, particularly a J-shaped relationship between sodium intake and cardiovascular mortality in women, which has not been extensively explored in previous guidelines. This study contributes to the existing body of knowledge by suggesting that while sodium reduction is essential, overly restrictive sodium intake in women with hypertension may also elevate CVD mortality risk, indicating that optimal sodium intake levels may differ by sex. These findings underscore the importance of considering individualized dietary recommendations for hypertensive women, which could potentially refine current clinical guidelines.

Several possible reasons could account for the association between low sodium intake and increased risk of CVD mortality. Reduced-sodium diet activates the sympathetic nervous system, which has been linked to a greater risk of CVD mortality (30, 31). Moreover, Sodium restriction can also increase insulin resistance directly (32, 33) or indirectly through an increase in sympathetic nerve activity (34, 35).

### Strength

This study has several strengths. Firstly, this study utilized the NHANES, a nationwide survey that has a large sample size and a good generalization of US adult. As a result, we were able to adjust for various covariates and improve the generalizability of the findings. Secondly, this study is the first to show a J-shaped relationship between dietary sodium intake and CVD mortality in hypertensive adults, which exists only in females and not in males. Sensitivity analysis in subjects without severe CVD limited the possibility of reverse causation.

### Limitation

This study does have several limitations. Firstly, Sodium intake was estimated by the single 24 h dietary recall method rather than gold-standard 24-h Urinary Sodium Excretion. In addition, sodium intake recorded at baseline may not reflected long term dietary exposure. Therefore, the identified threshold of 2.07 g/day, while statistically significant, may have limited direct applicability in clinical practice, where uniform dietary recommendations for salt intake restriction are typically advised for hypertensive patients. Secondly, causes of death were identified by linking NHANES-related mortality data from 2003 to 2012 with the National Death Index, using death certificate information. Although this method has been validated by the Centers for Disease Control and Prevention (CDC), the possibility of misclassification of causes of death cannot be entirely excluded. Thirdly, despite adjusting for a wide range of confounding variables in our analyses, the potential influence of unmeasured confounders remains a limitation.

## Conclusion

In summary, dietary sodium intake is non-linearly associated with CVD mortality in hypertensive patients. Hypertensive individuals with low and high sodium intakes were at increased risk of CVD mortality, though low sodium intake did not reach statistical significance. Further, we found a J-shaped association between dietary sodium intake and CVD mortality, only in female, but not in male. Large prospective studies are needed to further substantiate these finding.

## Data Availability

The raw data supporting the conclusions of this article will be made available by the authors, without undue reservation.

## References

[1] Mills KT Stefanescu A He J. The global epidemiology of hypertension. Nat Rev Nephrol. (2020) 16(4):223–37. 10.1038/s41581-019-0244-2 32024986 PMC7998524

[2] World Health Organization. WHO Guidelines approved by the guidelines review committee. In: Guideline: Sodium Intake for Adults and Children. Geneva: World Health Organization (2012).

[3] Arnett DK Blumenthal RS Albert MA Buroker AB Goldberger ZD Hahn EJ 2019 ACC/AHA guideline on the primary prevention of cardiovascular disease: a report of the American College of Cardiology/American Heart Association task force on clinical practice guidelines. Circulation. (2019) 140(11):e596–646. 10.1161/CIR.0000000000000678 30879355 PMC7734661

[4] Williams B Mancia G Spiering W Agabiti Rosei E Azizi M Burnier M 2018 ESC/ESH guidelines for the management of arterial hypertension. Eur Heart J. (2018) 39(33):3021–104. 10.1093/eurheartj/ehy339 30165516

[5] O'Donnell M Mente A Alderman MH Brady AJB Diaz R Gupta R Salt and cardiovascular disease: insufficient evidence to recommend low sodium intake. Eur Heart J. (2020) 41(35):3363–73. 10.1093/eurheartj/ehaa586 33011774

[6] Geleijnse JM Witteman JC Stijnen T Kloos MW Hofman A Grobbee DE. Sodium and potassium intake and risk of cardiovascular events and all-cause mortality: the Rotterdam study. Eur J Epidemiol. (2007) 22(11):763–70. 10.1007/s10654-007-9186-2 17902026 PMC2071962

[7] Kodjoe E. Low sodium intake and cardiovascular disease mortality among adults with hypertension. Int J Cardiol Cardiovasc Risk Prev. (2022) 15:200158. 10.1016/j.ijcrp.2022.200158 36573188 PMC9789348

[8] Tuomilehto J Jousilahti P Rastenyte D Moltchanov V Tanskanen A Pietinen P Urinary sodium excretion and cardiovascular mortality in Finland: a prospective study. Lancet. (2001) 357(9259):848–51. 10.1016/S0140-6736(00)04199-4 11265954

[9] Nagata C Takatsuka N Shimizu N Shimizu H. Sodium intake and risk of death from stroke in Japanese men and women. Stroke. (2004) 35(7):1543–7. 10.1161/01.STR.0000130425.50441.b0 15143292

[10] Umesawa M Iso H Date C Yamamoto A Toyoshima H Watanabe Y Relations between dietary sodium and potassium intakes and mortality from cardiovascular disease: the Japan collaborative cohort study for evaluation of cancer risks. Am J Clin Nutr. (2008) 88(1):195–202. 10.1093/ajcn/88.1.195 18614741

[11] He J Ogden LG Vupputuri S Bazzano LA Loria C Whelton PK. Dietary sodium intake and subsequent risk of cardiovascular disease in overweight adults. JAMA. (1999) 282(21):2027–34. 10.1001/jama.282.21.2027 10591385

[12] Raper N Perloff BP Ingwersen LA Steinfeldt LC Anand J. An overview of USDA’s dietary intake data system. J Food Compost Anal. (2004) 17:545–55. 10.1016/j.jfca.2004.02.013

[13] Levey AS Stevens LA Schmid CH Zhang YL Castro AF 3rd Feldman HI A new equation to estimate glomerular filtration rate. Ann Intern Med. (2009) 150(9):604–12. 10.7326/0003-4819-150-9-200905050-00006 19414839 PMC2763564

[14] Hu L Hu G Xu BP Zhu L Zhou W Wang T U-shaped association of serum uric acid with all-cause and cause-specific mortality in US adults: a cohort study. J Clin Endocrinol Metab. (2020) 105(1):dgz068. 10.1210/clinem/dgz068 31650159

[15] Cook NR Appel LJ Whelton PK. Sodium intake and all-cause mortality over 20 years in the trials of hypertension prevention. J Am Coll Cardiol. (2016) 68(15):1609–17. 10.1016/j.jacc.2016.07.745 27712772 PMC5098805

[16] Joosten MM Gansevoort RT Mukamal KJ Lambers Heerspink HJ Geleijnse JM Feskens EJ Sodium excretion and risk of developing coronary heart disease. Circulation. (2014) 129(10):1121–8. 10.1161/CIRCULATIONAHA.113.004290 24425751

[17] Singer P Cohen H Alderman M. Assessing the associations of sodium intake with long-term all-cause and cardiovascular mortality in a hypertensive cohort. Am J Hypertens. (2015) 28(3):335–42. 10.1093/ajh/hpu141 25159082 PMC4402347

[18] Thomas MC Moran J Forsblom C Harjutsalo V Thorn L Ahola A The association between dietary sodium intake, ESRD, and all-cause mortality in patients with type 1 diabetes. Diabetes Ccare. (2011) 34(4):861–6. 10.2337/dc10-1722 PMC3064042 21307382

[19] Ekinci EI Clarke S Thomas MC Moran JL Cheong K MacIsaac RJ Dietary salt intake and mortality in patients with type 2 diabetes. Diabetes Ccare. (2011) 34(3):703–9. 10.2337/dc10-1723 PMC3041211 21289228

[20] O'Donnell MJ Yusuf S Mente A Gao P Mann JF Teo K Urinary sodium and potassium excretion and risk of cardiovascular events. JAMA. (2011) 306(20):2229–38. 10.1001/jama.2011.1729 22110105

[21] O'Donnell M Mente A Rangarajan S McQueen MJ Wang X Liu L Urinary sodium and potassium excretion, mortality, and cardiovascular events. N Engl J Med. (2014) 371(7):612–23. 10.1056/NEJMoa1311889 25119607

[22] Stolarz-Skrzypek K Kuznetsova T Thijs L Tikhonoff V Seidlerová J Richart T Fatal and nonfatal outcomes, incidence of hypertension, and blood pressure changes in relation to urinary sodium excretion. JAMA. (2011) 305(17):1777–85. 10.1001/jama.2011.574 21540421

[23] Mente A O'Donnell M Rangarajan S Dagenais G Lear S McQueen M Associations of urinary sodium excretion with cardiovascular events in individuals with and without hypertension: a pooled analysis of data from four studies. Lancet. (2016) 388(10043):465–75. 10.1016/S0140-6736(16)30467-6 27216139

[24] He FJ Tan M Ma Y MacGregor GA. Salt reduction to prevent hypertension and cardiovascular disease: JACC state-of-the-art review. J Am Coll Cardiol. (2020) 75(6):632–47. 10.1016/j.jacc.2019.11.055 32057379

[25] Colpani V Baena CP Jaspers L van Dijk GM Farajzadegan Z Dhana K Lifestyle factors, cardiovascular disease and all-cause mortality in middle-aged and elderly women: a systematic review and meta-analysis. Eur J Epidemiol. (2018) 33(9):831–45. 10.1007/s10654-018-0374-z 29524110

[26] Matthews KA Crawford SL Chae CU Everson-Rose SA Sowers MF Sternfeld B Are changes in cardiovascular disease risk factors in midlife women due to chronological aging or to the menopausal transition? J Am Coll Cardiol. (2009) 54(25):2366–73. 10.1016/j.jacc.2009.10.009 20082925 PMC2856606

[27] Dubey RK Oparil S Imthurn B Jackson EK. Sex hormones and hypertension. Cardiovasc Res. (2002) 53(3):688–708. 10.1016/S0008-6363(01)00527-2 11861040

[28] Balafa O Kalaitzidis RG. Salt sensitivity and hypertension. J Hum Hypertens. (2021) 35(3):184–92. 10.1038/s41371-020-00407-1 32862203

[29] He J Gu D Chen J Jaquish CE Rao DC Hixson JE Gender difference in blood pressure responses to dietary sodium intervention in the GenSalt study. J Hypertens. (2009) 27(1):48–54. 10.1097/HJH.0b013e328316bb87 19145767 PMC2882679

[30] Darbar D Fromm MF Dellorto S Roden DM. Sympathetic activation enhances QT prolongation by quinidine. J Cardiovasc Electrophysiol. (2001) 12(1):9–14. 10.1046/j.1540-8167.2001.00009.x 11204091

[31] Hozawa A Ohkubo T Kikuya M Ugajin T Yamaguchi J Asayama K Prognostic value of home heart rate for cardiovascular mortality in the general population: the Ohasama study. Am J Hypertens. (2004) 17(11):1005–10. 10.1016/j.amjhyper.2004.06.019 15533725

[32] Petrie JR Morris AD Minamisawa K Hilditch TE Elliott HL Small M Dietary sodium restriction impairs insulin sensitivity in noninsulin-dependent diabetes mellitus. J Clin Endocrinol Metab. (1998) 83(5):1552–7. 10.1210/jcem.83.5.4835 9589654

[33] Feldman RD Schmidt ND. Moderate dietary salt restriction increases vascular and systemic insulin resistance. Am J Hypertens. (1999) 12(6):0–647. 10.1016/S0895-7061(99)00016-3 10371376

[34] Bjrntorp P Holm G Rosmond RJ. Hypothalamic arousal, insulin resistance and type 2 diabetes mellitus. Diabet Med. (1999) 16(5):373–83. 10.1046/j.1464-5491.1999.00067.x 10342336

[35] Mccarty MF. Elevated sympathetic activity may promote insulin resistance syndrome by activating alpha-1 adrenergic receptors on adipocytes. Med Hypotheses. (2004) 62(5):830–8. 10.1016/j.mehy.2003.11.007 15082116

