# Supplementary material for: Sex modifies association between dietary sodium intake and cardiovascular disease mortality among US adult with hypertension: a national population-based cohort
Source: Front Cardiovasc Med. 2024 Nov 28;11:1471647. doi: 10.3389/fcvm.2024.1471647 (PMC11634876; doi:10.3389/fcvm.2024.1471647)
Supplement: Supplementary file 1 [file Datasheet1.docx]

**Association between dietary sodium intake and** **Cardiovascular Disease Mortality Among US adult with hypertension: A National Population-Based Cohort**

Table S1: Hazard ratios of CVD mortality by sodium intake among hypertensive adults with no history of pre-existing CVD.

| Sodium intake | Person-years | Events | CVD mortality rate | HR (95% CI), P value | | |
| --- | --- | --- | --- | --- | --- | --- |
|  |  |  | (per year 1000 people) | Model 1 | Model 2 | Model 3 |
| **Per 1 g increase** | 110671.42 | 514 | 4.64 | 1.07 (1.01, 1.15) 0.027 | 1.07 (0.99, 1.16) 0.097 | 1.10 (0.99, 1.22) 0.074 |
| **Inflection point** |  |  |  | 2.07 | 2.07 | 2.07 |
| Sodium intake<2.07 | 25616.42 | 157 | 6.13 | 0.76 (0.57, 1.03) 0.073 | 0.79 (0.58, 1.07) 0.123 | 0.74 (0.50, 1.09) 0.132 |
| Sodium intake>2.07 | 85055.00 | 357 | 4.20 | 1.13 (1.05, 1.22) 0.001 | 1.12 (1.02, 1.23) 0.014 | 1.17 (1.04, 1.31) 0.009 |
| P for log likelihood ratio test |  |  |  | 0.024 | 0.046 | 0.046 |

*Note*: Model 1 was adjusted age,

Model 2 adjusted age, sex, race/ethnicity, education level, smoking status, alcohol using, diabetes, BMI, potassium intake;

Model 3 adjusted age, sex, race/ethnicity, education level, smoking status, alcohol using, diabetes, BMI, potassium intake, energy intake, antihypertensive drugs, glucose-lowering drugs, cholesterol-lowering drugs, SBP, DBP, eGFR, TC, HDL-C, triglyceride, ALT, AST, uric acid.

*Abbreviations*: BMI: body mass index; SBP, systolic blood pressure; DBP, diastolic blood pressure; eGFR, estimated glomerular filtration rate; TC, Total cholesterol, HDL-C, high density lipoprotein cholesterol; AST, aspartate aminotransferase; ALT, Alanine Aminotransferase;

Table S2: Hazard ratios of CVD mortality by sodium intake among hypertensive female with no history of pre-existing CVD.

| Sodium intake | Person-years | Events | CVD mortality rate | HR (95% CI), P value | | |
| --- | --- | --- | --- | --- | --- | --- |
|  |  |  | (per year 1000 people) | Model 1 | Model 2 | Model 3 |
| **Per 1 g increase** | 54659.25 | 239 | 4.37 | 1.04 (0.93, 1.16) 0.475 | 1.13 (0.99, 1.29) 0.075 | 1.16 (0.97, 1.38) 0.099 |
| **Inflection point** |  |  |  | 2.07 | 2.07 | 2.07 |
| Sodium intake<2.07 | 17132.83 | 102 | 5.95 | 0.61 (0.42, 0.90) 0.012 | 0.66 (0.44, 0.98) 0.041 | 0.53 (0.32, 0.88) 0.015 |
| Sodium intake>2.07 | 37526.42 | 137 | 3.65 | 1.18 (1.03, 1.35) 0.015 | 1.28 (1.10, 1.49) 0.002 | 1.39 (1.14, 1.70) 0.001 |
| P for log likelihood ratio test |  |  |  | 0.007 | 0.007 | 0.003 |

*Note*: Model 1 was adjusted age,

Model 2 adjusted age, race/ethnicity, education level, smoking status, alcohol using, diabetes, BMI, potassium intake;

Model 3 adjusted age, race/ethnicity, education level, smoking status, alcohol using, diabetes, BMI, potassium intake, energy intake, antihypertensive drugs, glucose-lowering drugs, cholesterol-lowering drugs, SBP, DBP, eGFR, TC, HDL-C, triglyceride, ALT, AST, uric acid.

*Abbreviations*: BMI: body mass index; SBP, systolic blood pressure; DBP, diastolic blood pressure; eGFR, estimated glomerular filtration rate; TC, Total cholesterol, HDL-C, high density lipoprotein cholesterol; AST, aspartate aminotransferase; ALT, Alanine Aminotransferase;

Table S3: Hazard ratios of CVD Mortality by sodium intake among hypertensive male with no history of pre-existing CVD.

| Sodium intake | Person-years | Events | CVD mortality rate | HR (95% CI), P value | | |
| --- | --- | --- | --- | --- | --- | --- |
|  |  |  | (per year 1000 people) | Model 1 | Model 2 | Model 3 |
| **Per 1 g increase** | 56012.17 | 275 | 4.91 | 1.04 (0.95, 1.13) 0.386 | 1.05 (0.94, 1.16) 0.396 | 1.09 (0.95, 1.24) 0.225 |
| **Inflection point** |  |  |  | 2.07 |  | 2.07 |
| Sodium intake<2.07 | 8483.59 | 55 | 6.48 | 0.91 (0.56, 1.47) 0.695 | 1.03 (0.62, 1.71) 0.908 | 1.15 (0.59, 2.22) 0.679 |
| Sodium intake>2.07 | 47528.58 | 220 | 4.63 | .05 (0.95, 1.16) 0.303 | 1.05 (0.93, 1.17) 0.434 | 1.08 (0.93, 1.26) 0.313 |
| P for log likelihood ratio test |  |  |  | 0.588 | 0.955 | 0.865 |

*Note*: Model 1 was adjusted age,

Model 2 adjusted age, race/ethnicity, education level, smoking status, alcohol using, diabetes, BMI, potassium intake;

Model 3 adjusted age, race/ethnicity, education level, smoking status, alcohol using, diabetes, BMI, potassium intake, energy intake, antihypertensive drugs, glucose-lowering drugs, cholesterol-lowering drugs, SBP, DBP, eGFR, TC, HDL-C, triglyceride, ALT, AST, uric acid.

*Abbreviations*: BMI: body mass index; SBP, systolic blood pressure; DBP, diastolic blood pressure; eGFR, estimated glomerular filtration rate; TC, Total cholesterol, HDL-C, high density lipoprotein cholesterol; AST, aspartate aminotransferase; ALT, Alanine Aminotransferase;


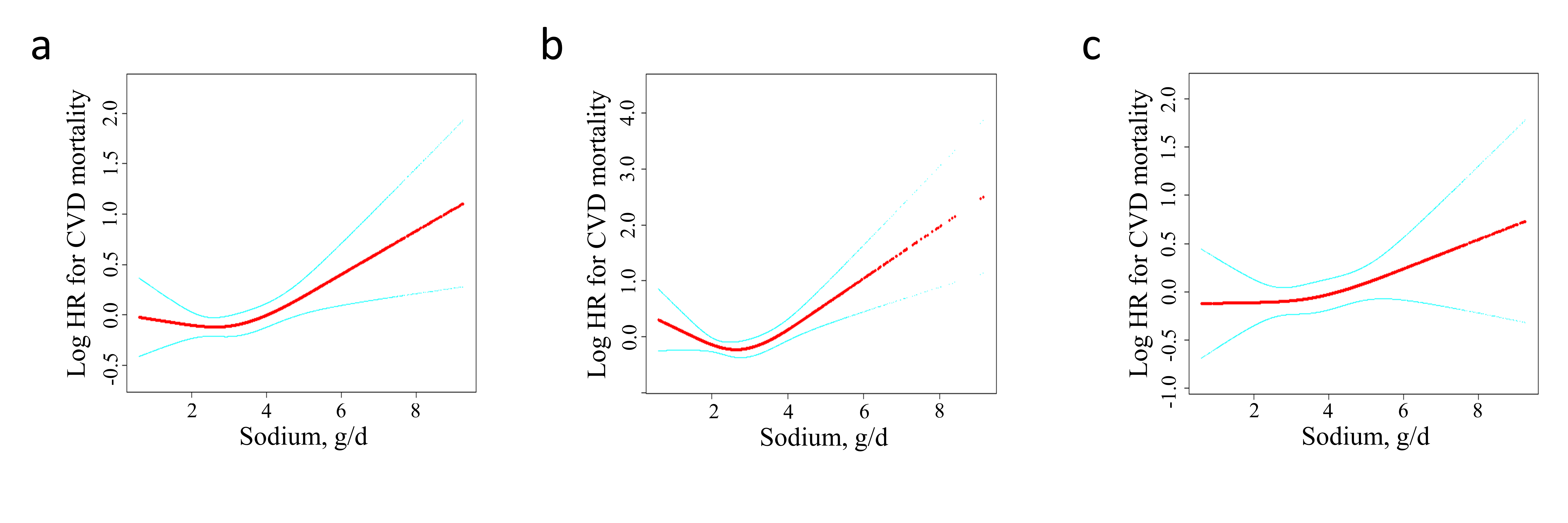


Figure S1: Dose-response analysis between dietary sodium intake and the risk of CVD mortality among (a) hypertensive adults with no history of pre-existing CVD (B) hypertensive females with no history of pre-existing CVD (C) hypertensive males with no history of pre-existing CVD

Adjustment factors included age, sex (only for the overall population), race/ethnicity, education level, smoking status, alcohol using, diabetes, BMI, potassium intake, energy intake, antihypertensive drugs, glucose-lowering drugs, cholesterol-lowering drugs, SBP, DBP, eGFR, TC, HDL-C, triglyceride, ALT, AST, uric acid.

*Abbreviations*: BMI: body mass index; SBP, systolic blood pressure; DBP, diastolic blood pressure; eGFR, estimated glomerular filtration rate; TC, total cholesterol, HDL-C, high density lipoprotein cholesterol; AST, aspartate aminotransferase; ALT, alanine aminotransferase;
